# Renal amyloidogenic leukocyte chemotactic factor 2 combined with IgA nephropathy: A case report

**DOI:** 10.1097/MD.0000000000029638

**Published:** 2022-07-22

**Authors:** Hongzhao Xu, Ye Jia, Xueyao Wang, Hui Wang, Jinyu Yu, Wu Hao

**Affiliations:** a Department of Nephrology, the first affiliated hospital of Jilin University, Changchun 130021, China; b Laboratory of Electron Microscopy, Peking University First Hospital, Beijing 100034, P.R. China; c Department of Pathology, the first affiliated hospital of Jilin University, Changchun 130021, China.

**Keywords:** amyloidogenic leukocyte chemotactic factor 2, IgA nephropathy, treatment

## Abstract

Amyloidogenic leukocyte chemotactic factor 2 (ALECT2) was recently considered as a new clinicopathologic type of amyloid, which frequently affects kidney in adults and results in different degrees of renal insufficiency and failure with or without proteinuria. Here, we present a case of combining LECT2-associated renal amyloidosis with immunoglobulin (Ig)A nephropathy.

**Patient concerns:**

A 71-year-old Chinese man presented with edema of both lower extremities.

**Diagnoses:**

There was pale eosinophilic material strongly positive for the Congo red stain in interstitium with demonstrated apple green birefringence under polarized light. Immunofluorescent stain was positive for IgA deposits (4+), IgG deposits (2+), C3 deposits (3+) within the mesangium and capillary wall. Immunohistochemistry was positive for κ (+), λ (2+) in mesangial area, and LECT2 (2+) in the interstitium. On electron microscopy, there were electron-dense deposits within mesangial area and subendothelial and randomly orientated and nonbranching fibrils 10 nm in size found in the interstitium areas. Liquid chromatography tandem mass spectrometry was performed on peptides extracted from Congo red-positive, microdissected areas of the paraffin-embedded kidney specimen. LECT 2-associated renal amyloidosis with IgA nephropathy was pathologically confirmed by renal biopsy.

**Interventions:**

Steroids (60 mg/d) were used to treat IgA nephropathy daily. Antihypertensive treatment was switched to an angiotensin-converting enzyme inhibitor.

**Outcomes:**

One year after diagnosis, creatine remained stable in the normal range, and 24-hour proteinuria decreased to 2.9 g.

**Lessons:**

To date, ALECT2 has still not been comprehensively investigated. The findings of this research provide insights for concurrent IgA nephropathy with ALECT2.

## 1. Introduction

Amyloidosis is a conformational disorder of protein folding characterized by caused by deposition of insoluble protein fibrils in multiple organs, which can be systemic or localized. Based on immunohistochemistry and immunofluorescence studies, there are more 30 different types of precursor proteins that can cause amyloidosis, including amyloid A amyloidosis, light chain amyloidosis, fibrinogen Aα chain, apolipoprotein A-I and apolipoprotein A-II, lysozyme, gelsolin, transthyretin and cystatin C types, etc.^[1]^ Renal amyloidosis is a frequent manifestation caused by the acellular Congo red-positive with typical birefringence pathologic deposition of amyloid fibrils within glomeruli and/or the interstitium, leading to nephrotic syndrome and end-stage renal disease. Recently, amyloidogenic leukocyte chemotactic factor 2 (ALECT2) has been recognized as a new type of renal amyloidosis.

ALECT2 was originally identified in a 54-year-old woman who presented with nephrotic syndrome in 2008 by Benson et al.^[2]^ Previous reports have elucidated that ALECT2 has an ethnic and regional divergence. ALECT2 was the third most common type of renal amyloidosis following light chain amyloidosis and amyloid A amyloidosis in Chinese patients from a single center,^[3]^ while also been reported predominantly in Hispanics of Mexican descent and mainly from the Southwest United States.^[4–6]^

To date, ALECT2 is still not yet been comprehensively investigated. In this report, we describe a case of LECT2 amyloidosis in kidney concurrent with immunoglobulin (Ig)A nephropathy, providing new insights into renal LECT2 amyloidosis.

## 2. Case report

A 71-year-old Chinese man was admitted to our department for edema of both lower extremities. He denied fever, vomit, and diarrhea. His past medical history was significant only for hypertension. There was no history of diabetes, rheumatological disorders, and no exposure to over-the-counter or herbal medicines. He had no familial history of renal diseases. Physical examination was unremarkable except for a blood pressure of 160/90 mm Hg and edema of both lower extremities.

Urinalysis showed blood (3+) and protein (4+), and microscopy revealed 244.8 red blood cells per high power field (HPF). Twenty-four-hour proteinuria was 19.8 g with no Bence Jones protein detected. Albumin decreased to 23.2 g/L (normal range [NR] 40–55) with NR of creatine, sodium, potassium, calcium, phosphate, bilirubin, and alkaline phosphatase. Immunoglobulins showed a mildly decreased IgG at 4.21 g/L (NR 8.6–17.4), but normal IgA and IgM levels. Serum and urine electrophoresis was negative for monoclonal proteins. Serologies including the hepatitis panel, antineutrophil cytoplasmic antibodies, antinuclear antibody, anti–double-stranded DNA antibody, thyroid function, tumor markers, and complements were within the NR. A renal ultrasound showed normal size kidneys.

**Figure 1.**
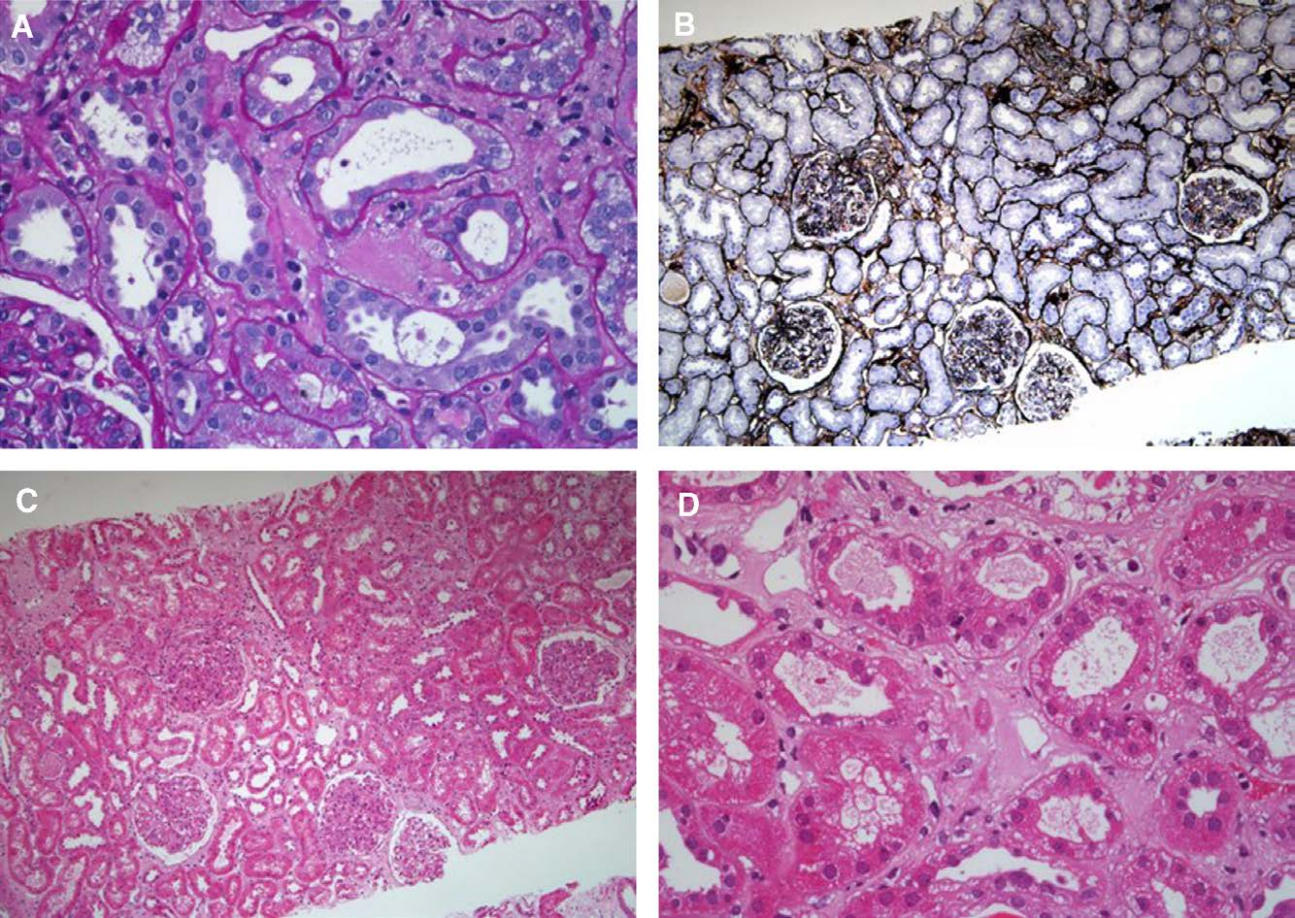
Light microscopy. Glomeruli showed an increase in mesangial cellularity and a mild increase in mesangial matrix with endocapillary proliferation and neutrophil infiltrate. In the interstitium there was fibrosis and surrounding areas of lymphocytes and monocytes infiltrate by (A) periodic acid-Schiff staining (×400), (B) PASM staining (×100). (C) Hematoxylin and eosin staining (×100).(D) Hematoxylin and eosin staining (×400). PASM = periodic Schiff-methenamine.

**Figure 2.**
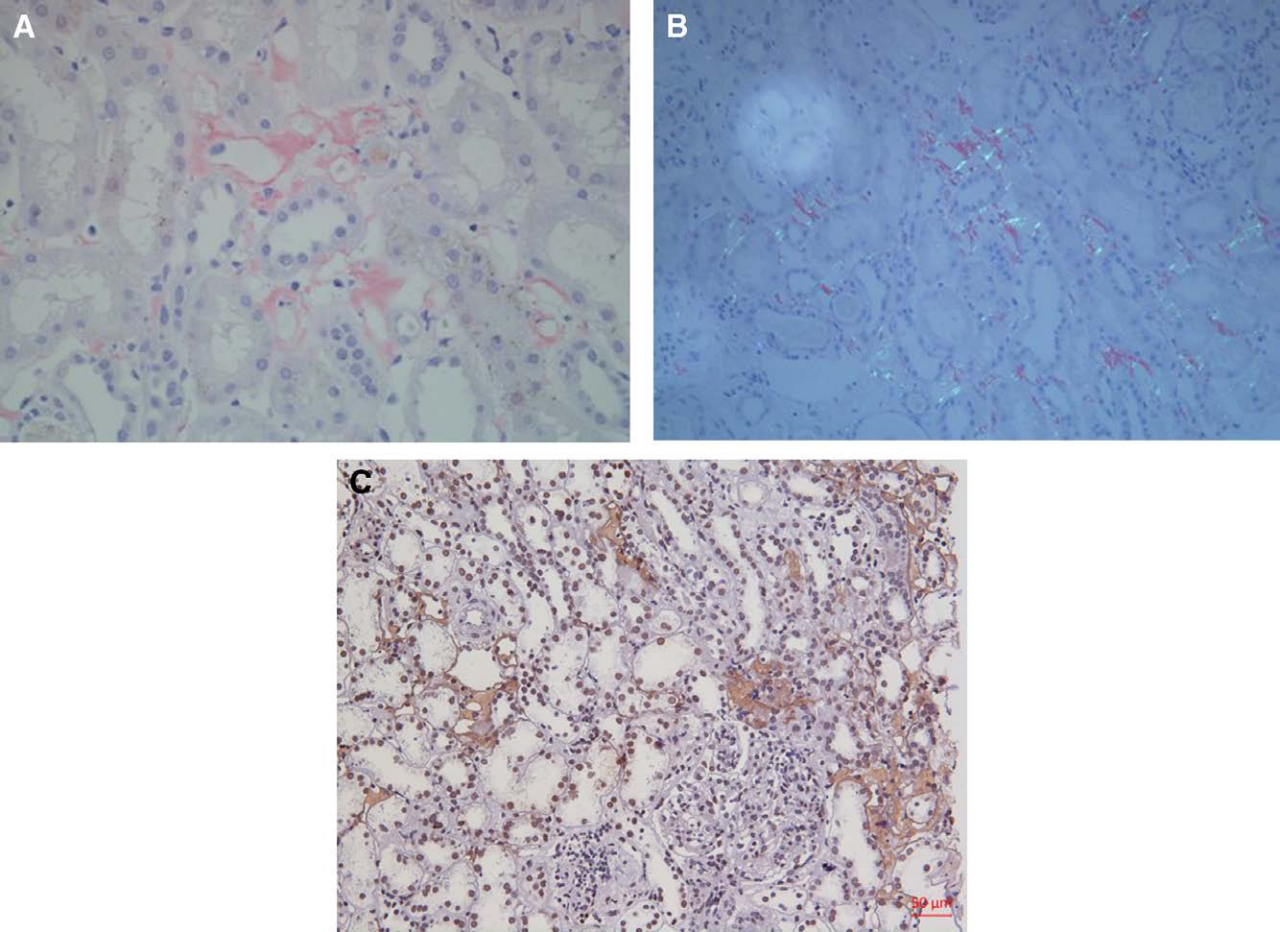
(A) Uptake of Congo red stain by interstitial amyloid deposits (×200). (B) showed typical birefringence under polarized light. (C) The amyloid deposits were specifically positive for LECT2 by IHC. IHC = immunohistochemistry, LECT2 = leukocyte chemotactic factor 2.

**Figure 3.**
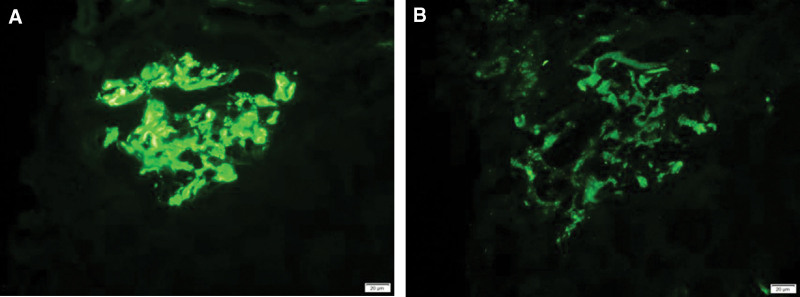
Immunofluorescent stain was positive for IgA deposits (4+) (A), C3 deposits (3+) (B) within the mesangium and capillary wall. IgA = immunoglobulin A.

**Figure 4.**
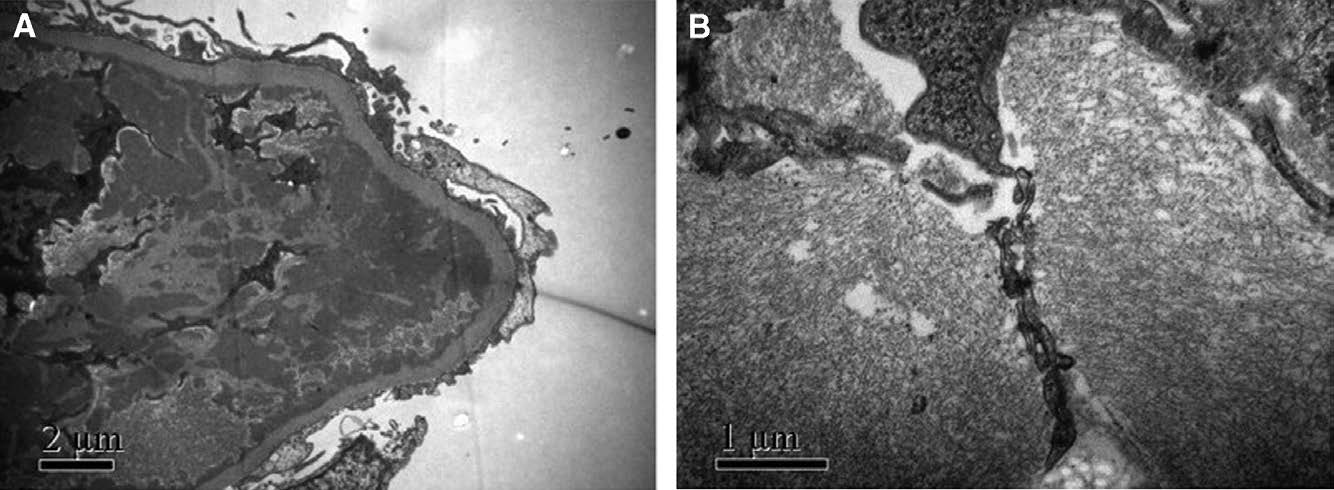
(A) (×10,000) and (B) (×30,000) Transmission electron photomicrograph of the interstitial region shows deposits of randomly arranged fibrils.

A renal biopsy was performed. Light microscopy revealed 39 glomeruli of which one was globally sclerosed, the glomeruli showed an increase in mesangial cellularity and a mild increase in mesangial matrix with endocapillary proliferation and neutrophil infiltrate. In the interstitium there was fibrosis and surrounding areas of lymphocytes and monocytes infiltrate (Fig. 1). There was pale eosinophilic material strongly positive for the Congo red stain in interstitium (Fig. 2A) with demonstrated apple-green birefringence under polarized light (Fig. 2B). Immunofluorescent stain was positive for IgA deposits (4+), IgG deposits (2+), and C3 deposits (3+) within the mesangium and capillary wall (Fig. 3). Immunohistochemistry was positive for κ (+), λ (2+) in mesangial area, and LECT2 (2+) in the interstitium (Fig. 2C). On electron microscopy, there were electron-dense deposits within mesangial area and subendothelial and randomly orientated and nonbranching fibrils 10 nm in size found in the interstitium areas (Fig. 4). Liquid chromatography tandem mass spectrometry (MS) was performed on peptides extracted from Congo red-positive, microdissected areas of the paraffin-embedded kidney specimen. This revealed LECT2-type amyloid deposition. The final diagnosis was IgA nephropathy with renal amyloidosis due to LECT2 amyloid deposition.

Steroids (60 mg/d) were used to treat IgA nephropathy. Antihypertensive treatment was switched to an angiotensin-converting enzyme inhibitor. One year after diagnosis, creatine has remained stable in the NR, and 24-hour proteinuria decreased to 2.9 g.

## 3. Discussion

Amyloidosis was characterized by pathologic deposits of specific fibrillary protein aggregates with distinct microscopic properties, particularly affinity for red-green birefringence in the presence of Congo red. ALECT2 was recently considered as a new clinicopathologic type of amyloid, which frequently affected kidney in adults and resulted in different degree of renal insufficiency and failure with or without proteinuria.^[7]^ The process appeared to be confined to the kidney; however, previous studies have discovered that extensive LECT2 deposition in the liver, heart, spleen, adrenal glands, colon, small intestine, gallbladder, lungs, bilateral ovaries, and uterus.^[8–10]^ There is current paucity of studies describing the etiology, pathogenesis, or prognosis of ALECT2.

*LECT2* gene encodes a versatile 16-kDa protein that acts as a chemotactic factor for the neutrophils migration and a hepatokine, which was correlated with the severity of obesity, hyperlipidemia, and insulin resistance.^[11–13]^ Moreover, it is also associated with rheumatoid arthritis, osteoarthritis, and cancers.^[14,15]^ *LECT2* gene has been mapped to chromosome 5q31.1–32 by genomic sequence analysis.^[13]^ The pathogenesis of ALECT2 might be due to the misfolding of LECT2 from genetic variations or mutations responsible for insoluble fibrils aggregation in different tissues.^[2,16]^ For instance, Ha et al^[17]^ discovered that loss of zinc together with the I40V mutation led to ALECT2. However, in our case, the patient refused to do genetic analysis for the LECT2.

On the basis of the findings of recent large renal biopsy series, the morphological pattern of amyloid distribution in all LECT2 specimens was similar with extensive involvement of all compartments of the kidney, including glomerular, mesangium, the inner layer of glomerular basement membrane, the renal cortical interstitium, medulla interstitium, and vascular walls.^[3,6,9,10,18]^ Immunohistochemistry studies can confirm the diagnosis, which showed strong staining for antibodies directed against LECT2 within the amorphous material. Recently, MS-based proteomics has been shown to be valuable for determining the nature and type of amyloid protein.^[19]^ Therefore, stained immunohistochemically and/or tandem MS were recommended for tissue from all cases of suspected ALECT2 amyloidosis.^[20]^ However, a previous study demonstrated that proteomic analysis of amyloidotic tissue by laser microdissection and MS may not be sufficient to diagnose ALECT2 amyloidosis in cases with little amyloid deposits and immunoelectron microscopy is a sensitive method for early diagnosis.^[3]^ Besides, the use of positron emission tomography computed tomography scan with florbetapir 18F could serve as a screening test for ALECT2 patients.^[21]^ It is significant to identify this type of amyloidosis to avoid initiation of unnecessary or possibly harmful therapies.

Based on observation in previous studies, high frequency of concurrent glomerular disease was discovered, ranging from 26%^[18]^ to 57%,^[3]^ including diabetic nephropathy, IgA nephropathy, membranous nephropathy, antineutrophil cytoplasmic antibodies–associated crescent glomerulonephritis, and focal segmental glomerulosclerosis.^[3,6,18]^ Moreover, podocytopathy, acute tubular injury, and interstitial nephritis should also be considered when a patient is diagnosed as ALECT with nephrotic syndrome.^[22,23]^ Up to now, there has been no investigation to illustrate comprehensively whether the concurrent ALECT2 amyloidosis and glomerular disease is pathogenetically correlated or only by chance, which needs further examination.

Currently, the effective treatment for ALECT2 is still not established caused by nonmutated precursor protein. Mejia-Vilet et al^[24]^ considered that donated kidneys with localized LECT2 amyloid deposits may be suitable for transplantation since amyloid deposits persist in the allograft appear not to interfere with allograft function. It seems kidney transplantation was a treatment option for ALECT2 patients with progressive disease.^[18]^ In our case, the patient was satisfied with the treatment he received since the proteinuria decreased with no apparent adverse events.

To date, ALECT2 has still not been comprehensively investigated. The findings of this research provide insights into concurrent IgA nephropathy with ALECT2.

## Author contributions

Conceptualization: Xu Hongzhao, Jia Ye, Wang Xueyao, Wu Hao.

Formal analysis: Xu Hongzhao, Jia Ye, Wu Hao.

Funding acquisition: Wu Hao. Investigation: Xu Hongzhao, Jia Ye.

Methodology: Wang Xueyao, Wanghui, Yu Jinyu. Project administration: Jia Ye, Wu Hao.

Writing – original draft: Xu Hongzhao. Writing – review & editing: Wu Hao.
